# Editorial: Heterogeneous Catalysis for Methane Activation

**DOI:** 10.3389/fchem.2022.962033

**Published:** 2022-06-23

**Authors:** Juanjuan Liu, Shihui Zou, Yun Liu, Jie Fan

**Affiliations:** ^1^ College of Materials and Environmental Engineering, Hangzhou Dianzi University, Hangzhou, China; ^2^ Key Lab of Applied Chemistry of Zhejiang Province, Department of Chemistry, Zhejiang University, Hangzhou, China; ^3^ Fritz-Haber-Institut der Max-Planck-Gesellschaft, Berlin, Germany; ^4^ Institute of Urban Environment, Chinese Academy of Sciences, Xiamen, China

**Keywords:** methane conversion, heterogeneous catalysis, C-H activation, selectivity, natural gas

Directly converting methane into value-added chemicals and fuels is a “dream reaction” in heterogeneous catalysis because it allows abundant natural gas and shale gas to be used as C1 building block for producing chemicals (Zou et al., 2021). The high molecular stability, however, makes it difficult to directly convert methane into desired chemicals in an economically attractive way. Despite the challenges, significant progress has been made recently in the selective activation of methane into methanol (Agarwal et al., 2017; Sushkevich et al., 2017; Jin et al., 2020), acetic acid (Shan et al., 2017; Tang et al., 2018), ethylene (Wang et al., 2017; Dong et al., 2022), and aromatics (Guo et al., 2014). With the ever increasing of oil-to-gas price ratio, some methane-based chemical production (e.g., ethylene production) is even expected to compete with oil-based technologies in around 20 years (Cruellas et al., 2019). For a better understanding of direct methane conversion through heterogeneous catalysis, we proposed this Research Topic and invited researches worldwide to contribute original research and review articles.

Direct methane conversion reactions can operate at both high temperature and low temperature. Thermocatalytic direct non-oxidative methane conversion (DNMC) and oxidative coupling of methane (OCM) are usually conducted at high temperatures (>900 K) as they require high temperature to activate methane on the catalyst surface and desorb methyl radicals into gas phase for following transformation (Zou et al., 2021). With the development in precision synthesis, advanced *in-situ* characterization, and comprehensive theoretical modelling, the knowledge on OCM and DNMC has advanced considerably. Guo et al. (2014) reported that single iron sites embedded in a silica matrix enable DNMC exclusively to ethylene and aromatics. Unprecedented methane conversion at 48.1%, ethylene selectivity at 48.4%, and total hydrocarbon selectivity exceeded 99% was achieved at 1363 K. Cheng et al. achieved stable and high methane conversion and low coke selectivity in Fe/SiO_2_ catalyzed DNMC by using SrCe_0.8_Zr_0.2_O_3−δ_ (SCZO) as “hydrogen transformer” to lower its local concentration, favor “soft coke” formation and mitigate the reverse reaction of DNMC. Zhou et al. investigated the activation processes of lanthanum-containing OCM catalysts by *in situ* X-ray photoelectron spectroscopy, X-ray diffraction, and online mass spectroscopy. They found that the activation of La_2_O_2_CO_3_ involved a migration to the surface followed by surface desorption while the activation of La(OH)_3_ showed three major phase change steps of the catalyst structure. Thum et al. synthesized phase-pure precursor materials for transition-metal-doped CaO and systematically investigated their performances in OCM. The results indicate that transition metal (i.e., Mn, Ni, and Zn) doping in low quantities can be applied to improve the catalytic performance of CaO, but the overall effect is limited. Combining *in situ* characterizations with theoretical studies, Qian et al. (2020) suggested single Mg_4c_^2+^ site as the most active sites for Li/MgO while Kiani et al. (2021) identified isolated, pseudotetrahedral, Na-coordinated WO_4_ surface sites as the active sites for Mn-Na_2_WO_4_/SiO_2_ catalyzed OCM reaction. In contrast to the considerable progresses in mechanism studies, no breakthrough in OCM performance was obtained. The uncontrollable homogeneous transformation of ∙ CH_3_ in the presence of O_2_ thermodynamically favors the production of CO_*x*_ and sets a theoretical upper bound on C_2_ yield (∼28%). Theoretic studies suggest that the limit can be broken only if catalysts play significant role in both heterogeneous generation of ∙ CH_3_ and their subsequent transformations (Arutyunov and Strekova, 2017). The latter is viewed as a “miracle” and has not been achieved till recently. Zou et al. (2021) demonstrated that 5 wt% Na_2_WO_4_/SiO_2_ (5NaWSi) can capture CH_3_ ∙ radicals desorbed from La_2_O_3_ and selectively convert them into C_2_ species on the catalyst surface. A bifunctional OCM catalyst system, which use La_2_O_3_ as the methane activation center and 5NaWSi as CH_3_· coupling center, exhibits much improved C_2_ selectivity and achieves a C_2_ yield up to 10.9% at 570°C. This study confirms the feasibility of surface coupling of CH_3_· and point in an exciting new direction for OCM studies.

Inputting external electric potentials or light irradiation as driving forces can break the thermodynamic barrier of C-H activation and facilitate direct methane conversion at low temperature. In this Research Topic, Januario et al. reviewed recent advances on heterogeneous photocatalysis for methane conversion under mild conditions into valuable products. The combination of metal and semiconductor is suggested to be a good strategy to develop more active photocatalysts. For example, Yu et al. (2020) developed an inspiring photochemical looping process for conversion of CH_4_ to C_2_H_6_ over Ag/H_3_PW_12_O_40_/TiO_2_, achieving a methane coupling selectivity of over 90%, a quantitative yield of ethane of over 9%, high quantum efficiency (3.5% at 362 nm) and excellent stability. Recently, an ethane production rate of over 5,000 μmol g^−1^ h^−1^ with 90% selectivity is achieved in a flow reactor using Au nanoparticle decorated ZnO/TiO_2_ hybrid as photocatalysts for oxidative coupling of methane (Song et al., 2021). In addition to low-temperature OCM and DNMC, considerable progresses have also been achieved in the selective oxidation of methane into oxygenated products at low temperature. Typical heterogeneous catalysts including AuPd nanoparticles (Agarwal et al., 2017; Jin et al., 2020), Rh single-atom catalysts (Shan et al., 2017; Tang et al., 2018), graphene-confined single Fe atoms (Cui et al., 2018), and metallocavitins (Shteinman).

At last, our guest editor team would like to acknowledge the valuable contribution of all the authors and referees. We hope the readers enjoy the research of direct methane conversion and pursue their efforts in this important area.
